# Obstructive sleep apnea syndrome in non-obese patients

**DOI:** 10.1007/s11325-021-02412-1

**Published:** 2021-07-29

**Authors:** Caterina Antonaglia, Giovanna Passuti

**Affiliations:** ASUITs, Pulmonology Unit, University Hospital of Cattinara, Strada di Fiume 447, 34149 Trieste, Italy

**Keywords:** OSAS in non-obese, Non-obese OSA anatomic factor, Sleep apnea pathophysiology in non-obese, OSAS without obesity

## Abstract

Obstructive sleep apnea syndrome (OSAS) is characterized by symptoms and signs of more than 5 apneas per hour (AHI) at polysomnography or 15 or more apneas per hour without symptoms. In this review, the focus will be a subgroup of patients: adult non-obese subjects with OSA and their specific features. In non-obese OSA patients (patients with BMI < 30 kg/m^2^), there are specific polysomnographic features which reflect specific pathophysiological traits. Previous authors identified an anatomical factor (cranial anatomical factors, retrognatia, etc.) in OSA non-obese. We have hypothesized that in this subgroup of patients, there could be a non-anatomical pathological prevalent trait. Little evidence exists regarding the role of low arousal threshold. This factor could explain the difficulty in treating OSA in non-obese patients and emphasizes the importance of a specific therapeutic approach for each patient.

## Introduction

OSAS (obstructive sleep apnea syndrome) is characterized by repeated collapses of the upper airways that result in a marked reduction (hypopnea) or complete interruption (apnea) of the airflow. These events are followed by phasic oxyhemoglobin desaturations, with consequent intermittent hypoxemia, sympathetic hyperactivation, and sleep fragmentation. They make the disease a risk factor for cardiovascular problems [1], diabetes [2], stroke [3], premature death [4], reduction of cognitive functions [5], and quality of life [6]. Instead, visceral obesity constitutes the main risk factor for OSAS [7]. In addition to anatomical factors like obesity, retrognathia, laxity of the soft palate, or macroglossia, many other factors have been involved in the pathogenesis of the disease [8]. These include genetic predisposition, smoking, alcohol consumption, and gender. Pathophysiology of OSA remains complex: it is clear that the anatomical predisposition factors are present in all patients (in 30% of patients without other factor). In 70% of cases, there are one or more associated non-anatomical pathophysiological factors. This is responsible for a different phenotype of the disease [9].

There are four pathophysiological factors involved in the pathogenesis [9]:

1. Anatomical factor (obesity, craniofacial conformations of reduced dimensions, laxity of the soft palate or macroglossia, which can lead to greater collapse of the upper airways in non-obese patients, etc.)
2. Instability of ventilatory control, also known as high loop gain
3. Neuromuscular inefficiency of the dilator muscles of the upper airways
4. Increased propensity for nocturnal awakenings due to respiratory stimuli, or a reduced awakening threshold, also known as low arousal threshold [9]

This paper explores the role of low arousal threshold (present in 30–50% of all patients with OSA) in non-obese OSA patients [10].

The diagnosis of OSAS is based on the presence of specific symptoms and signs with confirmation of the presence of sleep apnea, with an instrumental examination. The gold standard exam is the polysomnography (PSG), routinely indicated for the diagnosis of respiratory sleep disorders. However, PSG is expensive. An excellent, less expensive alternative is nocturnal portable monitoring (PM), which can be used for the diagnosis of OSAS in high-risk patients [11]. The diagnosis is given for an AHI ≥ 15, even in the absence of symptoms, or for AHI between 5 and 15, only in the presence of the associated symptoms described above. OSAS is classified as mild if AHI ≥ 5, but < 15, moderate, if AHI ≥ 15, but < 30, and severe, if AHI ≥ 30 [12].

The treatment of choice is the application of continuous positive air pressure (CPAP), which has been seen to reduce the risk of long-term mortality. In recent years, attention has increasingly shifted to a personalized therapeutic approach for these patients, depending on which pathophysiological factor prevails. CPAP may not be the right therapy for everyone. For some patients, other approaches such as mandibular advancement devices (MAD), maxillofacial surgery, bariatric surgery in morbid obesity, hypoglossal nerve stimulation, or a pharmacological approach with targeted therapies may be more appropriate than CPAP [13].

## Methods

This paper is a narrative review. We used MEDLINE with the following search criteria or keywords: “OSAS in non-obese,” “non-obese OSA anatomic factor,” “sleep apnea pathophysiology in non-obese,” “OSAS without obesity.”

Our selection criteria are composed primary of articles with epidemiology and pathophysiology items and also polygraphic parameters in adult non-obese patients with OSA.

## Epidemiology

The prevalence of OSA with a moderate to severe disorder (AHI > 15) is approximately 3–23% in women and 9–49% in men in middle-aged people [14]. The main risk factors associated with the presence of OSA are older age, male gender, large neck circumference, obesity, and reported snoring [15].

OSA was first observed and mentioned in 1936 by the English author Charles Dickens, in a character named Joe (fat boy) in the book *The Pickwick Papers.* Since then, the presence of the syndrome has been associated with obesity. Still today, many physicians look for OSA only in overweight individuals.

Obesity is a major risk factor for OSA [8]. In the severely obese, the prevalence of OSA ranges from 55 to 90% [7].

In overweight men (BMI 25–29.9), the prevalence of mild to severe SDB has been approximately twofold higher in elderly people (37% vs. 18%, respectively). Among overweight women, the prevalence has been approximately fivefold higher (20% vs. 4%, respectively). This suggests BMI has been more strongly related to prevalence of sleep-disordered breathing (SDB) in the younger age stratum [7]. Weight gain and loss have been consistently associated with increasing and decreasing severity of SBD [16].

Obesity is an important anatomical factor, but BMI is imperfect to categorize the obese patients because the main factor is fat deposition in the upper airway (which correlates with abdominal fat) and the abdominal fat itself causes the reduction of lung volume that may decrease longitudinal tracheal traction forces and pharyngeal wall tension [17].

Approximately 20% of adults with OSA are non-obese [14]. OSA in non-obese patients is usually less severe and less frequent. However, it is essential to identify these patients because they are four times more likely to develop hypertension than obese without OSA [18]. Non-obese patients are at risk for early atherosclerosis approximately 2.7 times more than obese patients without OSA, and this risk increases as the severity of the syndrome increases [19].

## Anatomic factors in non-obese OSA patients

Non-obese patients with OSA exhibit additional risk factors that may contribute development of obstructive apneas. Some factors are the same for obese and non-obese patients: macroglossia, retrognathia, soft palate tissue alteration, inflammation, and edema of the larynx (related to smoking or alcohol or gastroesophageal reflux). In non-obese OSA patients, the most studied risk factor is structural alteration of the skull.

Other studies have focused on bony/soft cranic and neck tissue abnormalities. In one study, Mortimore et al. [20] consider the neck fat deposition in three matched subject groups: non-obese, non-snoring control subjects, non-obese with OSA, and obese with OSA. The non-obese patients with OSA had an excessive fat deposition, particularly anterolateral to the upper airway, when compared with control subjects [20].

Sakakibara H et al. [21] comparing soft tissue abnormalities in obese and non-obese OSA patients, soft tissue abnormalities were shown to be more important in the obese group. Non-obese patients were shown to have a narrower facial anterior–posterior (A-P) distance and a narrower bony pharynx. These results showed a clear distinction between the obese and non-obese OSA patients in terms of the correlation between cephalometric measurements and the severity of apnea. In non-obese OSA patients, AHI was negatively correlated with the anterior cranial base length and the mandibular length [21].

All anatomic factors impacting upper airway collapsibility are measured with critical occlusion pressure (Pcrit) and defined as the endo-pharyngeal pressure associated with upper airway (UA) collapse. Pcrit is an important determinant, but abnormalities in non-anatomic traits are also present in most patients.

## Phenotypic traits in non-obese OSA patients. Do non-obese OSA patients have common clinical or polysomnographic features?

Quintas et al. [22] found a frequency of 70.5% and 22% of OSA in obese and normal weight patients, respectively. Normal weight patients were women, snorers, non-smokers, non-drinker, younger, and with a smaller neck and waist circumference. The most common reason for consultation in a sleep clinic was snoring without a significant hypersomnolence (low score of Epworth scale questionnaire). Authors notice a lower AHI, lower T90 (time of sleep with oxygen saturation under 90%), fewer desaturations per hour, and higher mean oxyhemoglobin saturation in normal weight patients than in other groups [22].

Kumar et al. [23] compared clinical and polysomnographic data between obese and non-obese patients with OSA. Their study showed that in OSA patients, higher BMI, male gender, neck circumference, and loud snoring were more prevalent in obese patients that in non-obese. Mild OSA (AHI < 15) was more prevalent in non-obese. Regarding comorbidities, hypertension was significantly more frequent in obese patients. Also diabetes and hypothyroidism were more prevalent in obese patients but in a nonstatistical way. OSA is an independent risk factor for diabetes. Regarding polysomnographic parameters in OSA non-obese, there was a significantly lower AHI, a minimum oxygen saturation above 90%, while respiratory effort related arousal was significantly higher [23].

Garg R et al. [24] showed the same clinical and polysomnographic parameters. They noticed that non-obese subjects were more likely to take sedatives for sleeping compared to their obese counterpart. Ghanem and Mahmood [25], with 102 non-obese patients with OSA in their study, found a less restful sleep or a sleep with more awakenings in this subgroup.

These findings showed that non-obese patients with OSA usually have a less severe disease regarding: AHI, the desaturations index, and comorbidity. Recognizing the syndrome in non-obese patients is important because there is an associated cardiovascular risk [19]. Also non-obese OSA patients are usually younger so an early detection and care could reduce long-term risk associated with the syndrome. Several authors support the idea that common clinical and polysomnographic features suggest a particular prevalent pathophysiological factor [26].

We reviewed and summarized in Table 1 the demographic, clinical, and polysomnographic characteristics and comorbidities in non-obese vs obese patients with OSAS in four principal papers [23, 24, 27, 28].

**Table 1:** Demographic, clinical, and polysomnographic characteristics and comorbidities in non-obese vs obese patients with OSAS

Study	Theerakittikul et al. 2019 [27]	Gray et al. 2017 [28]	Garg et al. 2012 [24]	Kumar et al. 2020 [23]
Obesity	BMI > 27.5 kg/m^2^	BMI > 30 kg/m^2^	BMI > 30 kg/m^2^	BMI > 27.5 kg/m^2^
Number of patients	123 non-obese (29.4%) (tot 418)	37 non-obese (25%) (tot 148)	36 non-obese (44.4%) (tot 81)	23 non-obese (44.4%) (tot 95)
Age (years)	59.14 ± 13.48, older than non-obese (n.s.)	47 ± 2, younger than obese 56 ± 2 (n.s.)	52.8 ± 7.19, older than obese (n.s.)	44.82 ± 12.47, younger than obese (n.s.)
Gender (percentage male)	63.4% (n.s.)	68% (n.s.)	75% (n.s.)	91.30% (*p* 0.048)
Neck circumference (cm)	13.94 ± 1.10 non-obese vs 15.71 ± 1.54 obese (*p* 0.001) (inches)	Missing data	15.81 ± 1.71 non-obese vs 16.10 ± 1.78 obese (inches)	36.10 ± 3.62 non-obese vs 39.21 ± 4.18 obese (*p* 0.001)
AHI (events/h)	69.31 ± 25.17 non-obese vs 88.26 ± 29.72 obese (*p* 0.064)	15 (11–23) non-obese vs 26 (20–52) obese (*p* > 0.005)	24.36 ± 12.17 non-obese vs 50.09 ± 29.49 obese (*p* < 0.001)	Mild OSA non-obese 39.13% vs 5.55% obese (*p* < 0.0001) Severe OSA was higher in obese 66.66% vs 30.43% non-obese (*p* 0.002)
ODI (events/h)	2.50 ± 3.55 non-obese vs 5.93 ± 6.07 in obese (< 0.01)	Missing data	30.63 ± 15.63 non-obese vs 48.32 ± 13.08 obese	Lowest oxygen saturation (%) > 90 08 (34.78) non-obese vs 11 (15.27) obese (*p* 0.041)
SatO2 mean value (%)	Missing data	Missing data	94.59 ± 2.4 non-obese vs 89.69 ± 13 obese (*p* < 0.001)	
Total sleep time with oxygen under 90%	3.08 ± 10.06 non-obese vs obese 10.83 ± 20.62 (*p* < 0.001)	Missing data		
ESS	*8.60* ± *5.16 non-obese vs 9.64* ± *5.41 obese (n.s)*	9 ± 1 vs 9 ± 1 (n.s.)	11.5 ± 3.27 non-obese vs 13.07 ± 5.60 obese (n.s.)	9.86 ± 4.59 non-obese vs 10.6 ± 4.47 obese(*p* 0.493)
CPAP therapy	98.65vs 11.17 (*p* < 0.001)	Missing data	Missing data	Missing data
Compliance with CPAP	85.1% use in non-obese vs 94.8% use in obese (*p* 0.002)	36% not use in non-obese vs 13% not use in obese (*p* 0.03)	Missing data	Missing data
Comorbidities	Diabetes mellitus and hypertension less common in non-obese group (statistically significant)	Atrial fibrillation, Hypertension less common in non-obese group (*p* < 0.005)	Missing data	Hypertension and snoring more common in obese group (statistically significant)

*n.s*. not statistically

## Pathophysiological traits in non-obese OSA patients

Non-anatomic pathophysiologic traits are particularly important in contributing to the presence of OSA and we know that 70% of patients have anatomical factors combined with one or more non-anatomical phenotypes [9]. The main non-anatomical factors are the following:

1. Upper airway muscle lower responsiveness during sleep
2. Low respiratory arousal threshold
3. Higher loop gain

How these and other pathophysiologic factors interact, with upper airway collapsibility and anatomy, ultimately determines the presence of OSA and its severity.

Edwards and Eckert in 2014 [10] have suggested that a low respiratory arousal threshold is present in 30–50% of patients with OSA, and is related to BMI, the Epworth scale, and AHI. In particular, a low arousal threshold is more common in patients with low AHI and a lower BMI. Individuals with a low arousal threshold usually wake up before a severe gas exchange abnormality (low SpO2) has developed. In these patients, both chronic sleep fragmentation and intermittent hypoxia have been implicated. A low arousal threshold predisposes to apnea through repeated awakenings: decreased sleep continuity and prevention of deeper more stable sleep (N3 sleep stage), excessive reductions in partial pressure of carbon dioxide with dynamic ventilatory instability, and decreased respiratory drive to the upper airway muscles [29].

Emma L. Gray et al. [28] found a higher proportion of non-obese patients with OSA with a low respiratory arousal threshold (86% non-obese vs. 60% obese, *p* < 0.001). The patients in this study had the same clinical and polysomnographic features of the non-obese patients in previous studies, in particular lower AHI, but also a polysomnographic particular pattern with AHI < 30 events/h, nadir SpO2 > 82.5%, and fraction of hypopneas > 58.3%. The authors suggest that the presence of two or more of the previous three-point scale detects patients with OSA with a low arousal threshold with a high sensitivity (80%) and specificity (88%) [10]. The other non-anatomical traits that cause OSA may also be common in non-obese patients, but the arousal threshold is the only factor that has been studied with a clinical score, in a non-invasive way.

The role of a low arousal threshold is also suggested by a low CPAP tolerance in this subgroup of patients [10]. Furthermore, other authors reported that non-obese subjects with OSA were more likely to take sedatives for sleeping as compared to obese patients [24, 30] and this could support a role of low arousal threshold in this subgroup of patients. We believe that further pathophysiological studies are needed to better clarify the role of low arousal threshold in OSA patients and in the non-obese subgroup. These findings are significant not only because we can recognize OSA in non-obese patients, usually younger than other patients, but also to consider a specific therapy for this subgroup of patients.

## Treatment in non-obese OSA patients

Non-obese OSA patients with a low arousal threshold have poorer adherence to CPAP therapy [28]. Gray et al. [28] showed that no alternative to CPAP treatment alone is the preferred treatment (as mandibular advancement devices, upper airway surgery, new hypoglossal nerve stimulation therapy, etc.). Non-anatomical interventions (e.g., non-myorelaxant sedatives) to increase the threshold for arousal, alone, or in combination with existing therapies (e.g. CPAP or oral appliances) may yield greater therapeutic success in this group of patients [9]. Nevertheless, further studies are needed to better define the role of the other pathophysiological traits and the best therapeutic approach in this subgroup of patients with OSA.

## Conclusion

Improved understanding of the pathophysiology of OSA in recent years provides an opportunity to develop individualized therapies based on subpopulations and mechanisms. Non-obese patients with OSA are a subgroup of individuals with clinical, polysomnographic, and pathophysiological features. The early recognition of the disease, in patients with fewer clinical markers, is essential because they are usually younger and treatment of OSA in these patients has the role of long-term cardiovascular prevention [31].

In non-obese adults with OSA, poor adherence to CPAP reaffirms the increasingly important role of a personalized therapy [13].
